# Importance of Pattern Standard Deviation of Humphrey 10-2 Visual Field to Evaluate Central Visual Function in Patients with Early-Stage Glaucoma

**DOI:** 10.3390/jcm12155091

**Published:** 2023-08-02

**Authors:** Hee Jong Shin, Si Eun Oh, Chan Kee Park, Hae-Young Lopilly Park

**Affiliations:** Department of Ophthalmology, Seoul St. Mary’s Hospital, College of Medicine, The Catholic University of Korea, Seoul 06591, Republic of Korea; shinhj01@naver.com (H.J.S.); sieeun5151@naver.com (S.E.O.); ckpark@catholic.ac.kr (C.K.P.)

**Keywords:** glaucoma suspect, normal tension glaucoma, perimetry, pattern standard deviation, pattern electroretinogram

## Abstract

To explore various parameters that can evaluate the central visual impairment in patients with early-stage glaucoma, we included patients into a study with central visual impairments with an MD value greater than −6.0 dB on the 24-2 VF test. A possible association between structural parameters acquired by OCT and functional parameters of VF and PERG was determined. A total of 70 eyes of patients with suspected glaucoma or NTG underwent VF, OCT, and PERG examinations. The patients were classified into two groups according to the MD of the 24-2 VF test. We used Pearson correlation analysis to evaluate the relationships between GCIPL thickness/RNFL thickness and visual functional parameters, such as PERG and perimetry. Linear regression analyses were conducted to evaluate the significant factors affecting the PSD of VF 10-2. In the low MD group, the P50 amplitude presented significant correlations (r = 0.346, *p* = 0.048) with GCIPL thickness. In the correlation analysis of the high MD group, it was found that only the PSD of 10-2 uniquely presented borderline significant correlations with GCIPL thickness (r = −0.327, *p* = 0.055), and no other functional parameter showed significant correlation. Univariate and multivariate analyses revealed that GCIPL thickness was significantly associated with a PSD of 10-2 VF (*p* < 0.001 and 0.013, respectively). Among various parameters, the P50 amplitude and 10-2 PSD demonstrated statistically borderline significant structure-function relationships with GCIPL thickness in early-stage glaucoma.

## 1. Introduction

As the life expectancy and proportion of the older population continue to increase, the number of patients diagnosed with glaucoma is also increasing. Early diagnosis of glaucoma has improved due to advances in medical accessibility, such as the development of test equipment and expansion of health checkups. However, as a neurodegenerative disease, glaucoma is impossible to fundamentally treat despite effective medical and surgical therapies to reduce intraocular pressure [1]. Owing to this limitation, progressive vision loss among patients with glaucoma is common, and glaucoma still ranks as the second most common cause of blindness in the world [2]. Most of the patients diagnosed in the early stages are considered to have a good prognosis because glaucomatous visual field (VF) damage generally occurs in the 10°–30° region of the VF [3]; in contrast, recent studies have reported that paracentral damage within the 10° region of the VF, including macular involvement, occurs in the early stage of glaucoma [4,5]. Even in patients with relatively small initial field defects, there is a considerable diminishment in vision-related quality of life when the damage involves the central VF [6]. Therefore, early diagnosis of central VF involvement in glaucoma is crucial.

In order to evaluate this central macular damage at an early stage, it is good to observe abnormal test points in the central 12 points of 24-2 VFs; however, studies have reported that the 10-2 VF test is necessary because the 24-2 VF test can miss the damage [7,8]. On the contrary, it has been reported that it is not helpful to perform the 10-2 VF at the same time as the 24-2 VF in patients with early glaucomatous damage [9]. There has also been a study showing no significant difference of sensitivity when using pattern standard deviation (PSD) values for detecting central visual field abnormalities in early glaucoma patients between central 12 locations of the 24-2 VF and entire 10-2 VF [10]. Several studies have suggested that glaucomatous eyes with any abnormal 24-2 VF point in the central 10° region that correlates to macular ganglion cell–inner plexiform layer thinning deserves attention to determine early glaucomatous changes by performing a 10-2 VF test [11,12]. Recently, the SITA-faster 24-2c VF test, which adds ten additional test points derived from test locations that are commonly affected in glaucoma within the central 10° from fixation, has also proven its usefulness in evaluating central visual function [13,14]. In addition to visual field tests, pattern electroretinogram (PERG) also helps detect initial damage in the central VF region. It has been suggested that there is a disease stage in which dysfunction of retinal ganglion cells (RGCs) precedes cellular and axonal loss, resulting in functional losses in the presence of a normal structure, and can only be detected by PERG [15]. PERG can detect dysfunction; however, live RGCs [16] allow the early diagnosis of glaucoma.

To date, the structure-function relationship, especially in the central macular region, has not been sufficiently investigated. Mohammadzadeh et al. reported that correlations between central structural and functional rates of change were weak to fair in patients with central damage [17]. Although several previous studies have reported a strong association between macular structure and central visual function in advanced glaucoma [18,19], it has not been well reported whether this association can also be applicable in early-stage glaucoma. Hood et al. reported a good agreement between structural and functional damage, even in eyes with confirmed early glaucomatous damage, if both 24-2 and 10-2 VFs are obtained and abnormal locations on the VFs are compared to those observed on optical coherence tomography (OCT) macular and disc scans [20]. However, we still need to define functional parameters to detect early functional changes in the central macular region that have a better correlation with the structural parameters.

In this study, we aimed to explore various parameters that can evaluate central visual impairment in patients with early-stage glaucoma, including eyes in the preperimetric stage, according to the 24-2 VF test. A possible association between structural parameters acquired by OCT and functional parameters of VF and PERG was determined.

## 2. Materials and Methods

### 2.1. Participants

This cross-sectional study was approved by the Institutional Review Board of the Catholic University of Korea, Seoul, Republic of Korea, and was performed according to the tenets of the Declaration of Helsinki. A total of 70 eyes of patients with suspected glaucoma or normal-tension glaucoma (NTG) who satisfied the inclusion criteria at the Glaucoma Clinic of Seoul St. Mary’s Hospital between March 2022 and October 2022 were included. Informed consent was obtained from all participants.

Glaucoma suspects are defined as individuals with clinical findings or risk factors that may increase the likelihood of developing glaucoma, including high intraocular pressure (IOP) and abnormalities of the optic disc or retinal nerve fiber layer (RNFL). Glaucoma suspects with IOP within the normal range but with suspicious optic disc or RNFL findings are referred to as NTG suspects.

Patients were included if they had a best-corrected visual acuity >20/30 or better, a mean deviation value greater than −6 dB on 24-2 standard automatic perimetry (SAP), an open angle, and an axial length less than 28 mm. Patients were excluded if they had a history of uveitis, retinal diseases such as retinal vein obstruction, macular degeneration, and diabetic retinopathy, or a history of intraocular surgery except for uncomplicated cataract extraction. Patients with any optic nerve-related disease besides glaucoma and/or a history of systemic or neurological diseases that might affect VF or PERG were excluded. When both eyes fulfilled the inclusion criteria, one eye per individual was randomly selected for this study.

### 2.2. Measurements

All participants underwent complete ophthalmic examinations, including slit-lamp examination, Goldmann applanation tonometry, gonioscopy, central corneal thickness measurement, axial length biometry (IOLMaster; Carl Zeiss Meditec, Dublin, CA, USA), and dilated fundus biomicroscopy. 

### 2.3. Optical Coherence Tomography

Circumpapillary RNFL thickness and ganglion cell/inner plexiform layer (GCIPL) thickness were measured using Cirrus spectral-domain optical coherence tomography (SD-OCT, version 6.0; Carl Zeiss Meditec, Dublin, CA, USA). Detailed descriptions of the GCIPL or RNFL thickness have been previously described [21,22]. Only well-focused OCT images with signal strengths >6 were included. 

### 2.4. Pattern Electroretinogram

The electrophysiological test results were recorded using a commercial electroretinogram (ERG) stimulator (Neuro-ERG, Neurosoft, Ivanovo, Russia) by a trained examiner. The participants were seated in front of a display in a semi-dark room with a constant background illumination of 50 lx and had full optical correction according to their refraction before the examination. Two 35-mm Ag/AgCl skin electrodes were attached to the lower eyelids, with two ground electrodes in both earlobes. The visual stimulus was a checkerboard pattern with a mean luminance of 300 cd/m^2^ and contrast between black and white squares of 98%. The patterns on display were reversed in the counterphase at 4 Hz at a 60 cm distance from the patients. Black-and-white checkerboards with a check size of 1.81° were displayed on a 24-inch monitor with a 48 × 33 degree visual angle. All participants were instructed to focus intensely on the red fixation target at the center of the monitor screen. A detailed description of the examination is provided in our previous study [23,24]. The amplitudes of P50 and N95 were measured. The P50 amplitude was determined as the height from the trough of N35 to the peak of the P50. The amplitude of the N95 was measured from the P50 peak to the N95 trough.

### 2.5. VF Testing

Standard automatic perimetry using both 24-2 and 10-2 tests was performed by the SITA program (Humphrey Visual Field Analyzer; Carl Zeiss Meditec Inc., Dublin, CA, USA). Both 10-2 and 24-2 tests used the Swedish Interactive Thresholding Algorithm (SITA) standard strategy after refractive correction with a Goldmann size III target and background luminance (31.5 asb). All 10-2 and 24-2 VF tests were required to have fixation losses, false positives, and false negatives of ≤25%.

### 2.6. Definition of Mean Deviation (MD) and Pattern Standard Deviation (PSD)

The MD is the average value of all test points in the total deviation plot, which is based on the deviation from the age-matched normal values. Participants who can observe dimmer stimuli than others of similar age and race will have positive MD values, while participants who require brighter stimuli will have negative MD values. Although MD is a useful indicator of total depression in visual field sensitivity that shows a linear change according to glaucoma progression [25], generalized depression can result not only from glaucoma but also from media opacity, such as cataract, or decreased retinal sensitivity, such as high myopia [26,27]. The PSD values are calculated based on the variation from the normal age-corrected hill of vision involving the total deviation plot. PSD is a metric that indicates the difference in the sensitivity of adjacent tested points. In patients with glaucoma, as irregular depression of visual field sensitivity progresses, the PSD values increase. However, as visual field damage progresses to the point of causing an overall reduction in sensitivity, the PSD values decrease. Hence, the PSD is considered an inappropriate parameter for determining the stage of glaucoma [28,29].

### 2.7. Classifying into High and Low MD Groups

The patients were classified into two groups according to the MD of the 24-2 VF test. Half of the patients with a relatively high SAP 24-2 MD > −1.67 dB were assigned to the high MD group. The other half of the patients were assigned to the low MD group.

### 2.8. Creation of Threshold-Sensitive Points (Total and Center)

**Figure 1:**
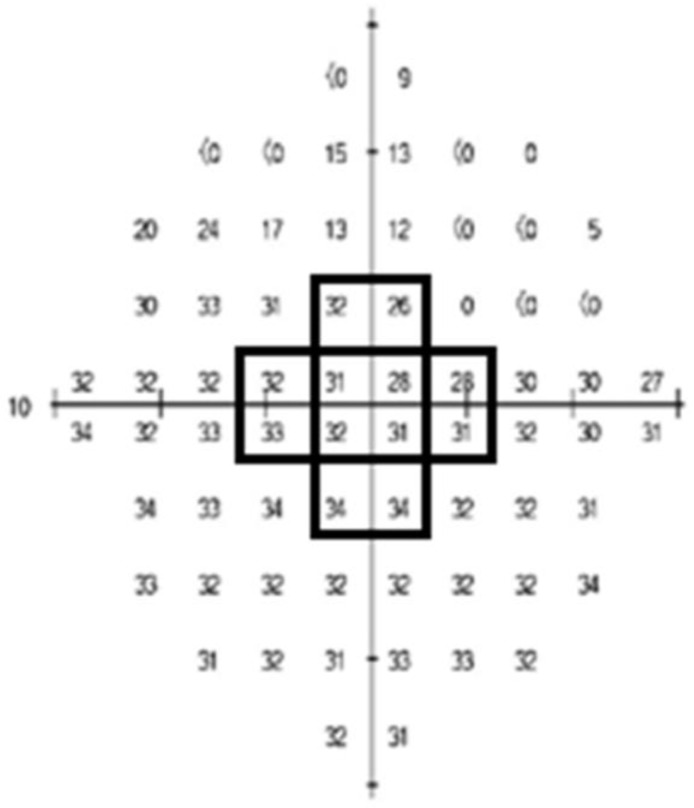
The number averaged by summing the 12 points in the black squares; the central innermost 4° of 10-2 Humphrey visual field test was named “Threshold sensitive points (center)”.

One of the most used standard automated perimetry programs for glaucoma, Humphrey 24-2 VF, includes a total of 54 test points (including two points for physiologic blind spots) that are 6° apart. However, 24-2 VF has only 12 test points within 10° of fixation and therefore lacks detailed spatial information in this region. The 10-2 VF examines the central 10° of the VF with 68 test points 2° apart. Therefore, the 10-2 VF may perform better than the 24-2 VF in detecting subtle changes in glaucomatous VF defects within the central 10°. We calculated an average of 68 values of the map of the threshold sensitivity in the 10-2 VF and named it “Threshold sensitive points (total),” which could represent the central visual function. In addition, we created a new parameter “Threshold sensitive points (center)” by calculating an average of 12 test points located in the central innermost 4° of 10-2 VF (Figure 1).

### 2.9. Statistical Analysis

All data are presented as the mean ± standard deviation. Student’s *t*-test and chi-square test were used to compare the characteristics and results of OCT, perimetry, and ERG between the low and high MD groups. We used Pearson correlation analysis to evaluate the relationships between GCIPL thickness/RNFL thickness and visual functional parameters, such as PERG and perimetry, by grouping participants into low and high MD and to calculate the correlation coefficients between 10-2 MD/PSD and other perimetry parameters and PERG results. Linear regression analyses were conducted to evaluate the significant factors affecting the PSD of VF 10-2. All statistical analyses were performed using the tidyverse, ggplot2, moonBook packages of R (version 4.2.1), and R Studio (version 2022.7.1.554) software; *p* < 0.05 was considered statistically significant.

## 3. Results

Seventy eyes of 70 patients who met the eligibility criteria underwent VF, OCT, and PERG examinations. Table 1 presents the baseline characteristics of the participants. The mean age was 52.91 ± 15.06 years and the MD of the 24-2 VF test was -2.15 ± 2.32 dB.

Half of the patients with relatively high MD were classified into the high MD group and another half with relatively lower MD were classified into the low MD group. Table 2 shows comparisons of the characteristics and demographic features between the two groups; mean age, male-to-female ratio, axial length, and central corneal thickness did not differ significantly. The mean RNFL/GCIPL thickness and N95 amplitude of the PERG were lower in the low MD group than that in the high MD group. Visual field parameters were worse in the low MD group than that in the high MD group (all *p* < 0.05, Table 2).

We analyzed the correlations between the average RNFL thickness and functional parameters of 24-2 VF, 10-2 VF, and PERG (Table 3). 

In all participants, there were significant correlations between the MD of 24-2 VF (r = 0.356, *p* < 0.001), PSD of 24-2 VF (r = −0.269, *p* = 0.024), PSD of 10-2 VF (r = −0.250, *p* = 0.036), and average RNFL thickness. Correlations between the average GCIPL thickness and functional parameters were also evaluated. In all participants, there were significant correlations in the MD of 24-2 VF (r = 0.240, *p* = 0.048), and all parameters measured from SITA 10-2 including the total and central threshold sensitive points. Additionally, the P50 amplitude from the PERG showed borderline significance (r = 0.237, *p* = 0.052). However, none of the functional parameters showed significant correlations with RNFL thickness when analyzed separately in the high and low MD groups (Table 4). 

In the low MD group, the results showed a similar pattern, except that the P50 amplitude presented additional significant correlations (r = 0.346, *p* = 0.048) with GCIPL thickness. In the correlation analysis of the high MD group, it was found that only the PSD of 10-2 uniquely presented borderline significant correlations with GCIPL thickness (r = −0.327, *p* = 0.055), and no other functional parameter showed significant correlation. Additionally, we analyzed whether the PSD of the 10-2 VF had a significant correlation with other functional parameters and showed a significant correlation with other VF parameters, however, not with parameters from the PERG (Table 5).

Table 6 presents the results of the linear regression analysis conducted to determine the factors associated with a PSD of 10-2 VF. 

With the total participants, univariate analysis revealed that GCIPL thickness, PSD of 24-2 VF, MD of 10-2 VF, and threshold-sensitive points of center and total were significant factors. (*p* < 0.001) In the multivariate analysis using stepwise regression, axial length, GCIPL thickness, MD, and PSD of 24-2 VF, MD of 10-2 VF, central threshold sensitive points were significantly associated with PSD of 10-2 VF (*p* < 0.01). Univariate and multivariate analyses revealed that GCIPL thickness was significantly associated with a PSD of 10-2 VF (*p* < 0.001 and 0.013, respectively). Additionally, linear regression analysis was performed on the P50 amplitude; however, it was not included in this study because no significant related parameters were found.

## 4. Discussion

Glaucoma is an incurable neurodegenerative disorder characterized by selective, progressive, and irreversible degeneration of RGCs and the optic nerve. Therefore, early diagnosis and appropriate treatment of glaucoma are crucial. Central visual function is an important part of the patient’s quality of life, and there are patients with central damage even in the early stages; therefore, it is important to diagnose early and comprehend the progress and treat them. As there are no global standards for diagnostic testing of glaucoma, diagnosis is generally based on characteristic changes in structural and functional testing. In general, VF tests, typically with a 6° grid (24-2 or 30-2 pattern), are used for functional tests, and OCT tests are used for structural tests. In patients with early glaucoma, there were reports that the 10-2 VF test was also useful; therefore, it was included in this study. Additionally, PERG helps detect early glaucomatous damage; therefore, it was included as a test to check for functional changes [16,24].

This study aimed to identify useful functional measures in the early stages of glaucoma, even in the suspected or preperimetric stage, in order to detect central functional changes that correlate with structural measures. The values of P50 and N95 amplitudes were measured using the PERG test, and the MD and PSD values were measured as VF tests. In addition, to determine whether the threshold sensitivity of the 10-2 VF test can be used as a parameter for central macular function, new values averaged for a total of 68 and central 12 points were measured. RNFL and GCIPL thicknesses were measured using OCT as indicators to identify structural damage, and their association with functional parameters was examined by dividing them into the entire patient and high and low MD groups. It was found that the low-MD group had significantly deteriorated structural and functional parameters (Table 2).

Since the study population had early glaucoma (mean MD of the 24-2 VF, −2.15 dB), it may be difficult to obtain strong significant correlations between functional and structural parameters. However, we showed that even in the early stage, patients with glaucoma show structure-function relationships in the central macular region since MD of 24-2 VF, threshold sensitivity, MD, and PSD of 10-2 VF have a significant correlation with GCIPL thickness, but not all with RNFL thickness. The fact that 10-2 VF test, which evaluates the central visual function, is highly related to GCIPL thickness is considered to show the structure-functional relationship well.

When we classified patients according to the MD of 24-2 VF, only the PSD of 10-2 VF showed borderline significance with GCIPL thickness in patients with high MD (mean MD of 24-2 VF, −0.3 dB). This indicates that very early functional changes in the central macular region may affect the PSD of 10-2 VF, even before the change in PERG parameters indicates RGC dysfunction. Threshold sensitive points of the central 12 points on 24-2 VF also showed similar results with PSD of 10-2 VF. However, this parameter is not automatically provided by the machine and it did not show statistical significance in the High MD group. Therefore, we think it may be important to use PSD of 10-2 VF to evaluate central function in early-stage glaucoma since it was the only parameter showing significant association with GCIPL thickness in preperimetric glaucoma.

Some studies have reported which indicators (MD or PSD) are more useful for evaluating disease progression. Gardiner et al. reported that PSD is significantly less predictable than MD and may also be a poorer predictor of subsequent change [30]. However, the superiority of MD over PSD in that pattern deviation analysis may underestimate the progression was reported [31], although that study did not look at MD and PSD directly. A possible explanation is that PSD is a measure of the spread of sensitivity values in the field, rather than an average (as with MD), and such measurements may be more affected by measurement noise. Contrary to previous studies, there was also a study that showed that VFI analysis based on pattern deviation seems to be more accurate than MD analysis for determining the rate of progression in patients without significant lens opacity [32]. PSD measures irregularity by summing the absolute value of the difference between the threshold value for each point and average visual field sensitivity at each point (equal to the normal value for each point + the MD). Visual fields with age-normal sensitivity at each point will have a PSD of zero, as will visual fields in which each point is uniformly depressed from the age-normal value. Thus, the largest PSD was registered for focal and deep visual field defects. Both near-normal and severely damaged visual fields had a low PSD. Unlike MD, which measures the degree to which it can respond to dimmer stimuli compared to similar age and race, PSD can detect a very small amount of initial change because it reflects the difference between the surrounding parts in the individual measurement. 

Additionally, N95 amplitude has been shown to change in glaucoma and glaucoma suspects [24,33]. Park et al. reported that the N95 amplitude was significantly correlated with GCIPL thickness in patients with early glaucoma, while visual field performance showed no correlation with GCIPL thickness [34]. However, only the P50 amplitude showed a significant correlation with GCIPL thickness in our study with early-stage glaucoma when no other parameters from 24-2 VF showed a correlation. This may suggest that early macular involvement changes the non-spiking activity between the soma/dendrites of RGC and bipolar cells.

Additionally, although it has been confirmed that there is no significant correlation, this is considered valuable as the first comparative study on the relationship between 10-2 VF parameters and PERG amplitudes, while previous studies have compared 24-2 VF and PERG.

The limitations of our present study include the small sample size and small number of tests. Therefore, larger studies should be undertaken in the future to determine the structure-function relationship present more precisely when detecting glaucoma progression. Additionally, the study evaluated 70 patients with early glaucoma (average age 52.91) and most of them had no lens opacity; therefore, a selection bias was considered in general patients.

## 5. Conclusions

We assessed the relationship between the functional parameters obtained from 10-2 VF, 24-2 VF, pattern ERG, and structural parameters obtained from Cirrus OCT in patients with early glaucoma. Although most functional parameters showed no significant association, the P50 amplitude and 10-2 PSD demonstrated statistically borderline significant structure-function relationships with GCIPL thickness. Given the importance of the central VF in patients’ quality of life, we suggest examining the PSD of 10-2 VF and the P50 amplitude of PERG to evaluate early macular involvement in very early-stage glaucoma.

## Figures and Tables

**Table 1:** Baseline demographics and ocular characteristics.

Variables	Description
Age (y)	52.91 ± 15.06
Female, no. (%)	35 (50%)
Axial length (mm)	24.98 ± 2.20
Central corneal thickness (μm)	538.27 ± 42.39
Average pRNFL thickness (μm)	82.29 ± 9.50
Average mGC/IPL thickness (μm)	74.30 ± 8.33
MD of VF 24-2 (dB)	−2.15 ± 2.32
PSD of VF 24-2 (dB)	3.32 ± 2.53
MD of VF 10-2 (dB)	−1.76 ± 2.99
PSD of VF 10-2 (dB)	2.51 ± 2.93
Threshold sensitive points (center)	32.47 ± 3.38
Threshold sensitive points (total)	31.23 ± 2.93
P50 amplitude of PERG	3.12 ± 1.31
N95 amplitude of PERG	5.35 ± 1.47

pRNFL, peripapillary retinal nerve fiber layer; mGC/IPL, macular ganglion cell-inner plexiform layer; VF, visual field; MD, mean deviation; PSD, pattern standard deviation; dB, decibel; PERG, pattern electroretinogram. Data are mean ± standard deviation unless otherwise indicated.

**Table 2:** Baseline demographics and ocular characteristics of High MD group and Low MD group patients.

Variables	High MD Group	Low MD Group	*p* Value
Age (y)	51.7 ± 14.2	54.2 ± 16.0	0.489 *
Female, no. (%)	20 (57.1%)	20 (57.1%)	1.000 ^†^
Axial length (mm)	24.9 ± 1.9	25.4 ± 2.4	0.447 *
Central corneal thickness (μm)	547.6 ± 43.9	529.6 ± 40.2	0.094 *
Average pRNFL thickness (μm)	85.8 ± 8.8	78.7 ± 8.9	**0.001** *
Average mGC/IPL thickness (μm)	76.3 ± 6.0	72.3 ± 9.8	**0.046** *
MD of VF 24-2 (dB)	−0.3 ± 1.0	−4.0 ± 1.8	**0.000** *
PSD of VF 24-2 (dB)	2.0 ± 1.0	4.6 ± 2.9	**0.000** *
MD of VF 10-2 (dB)	−0.6 ± 1.4	−2.9 ± 3.7	**0.001** *
PSD of VF 10-2 (dB)	1.7 ± 1.7	3.3 ± 3.6	**0.022** *
Threshold sensitive points (center)	33.5 ± 1.8	31.5 ± 4.2	**0.012** *
Threshold sensitive points (total)	32.3 ± 1.6	30.1 ± 3.5	**0.001** *
P50 amplitude of PERG	3.3 ± 1.5	2.9 ± 1.1	0.248 *
N95 amplitude of PERG	5.7 ± 1.7	5.0 ± 1.1	**0.047** *

pRNFL, peripapillary retinal nerve fiber layer; mGC/IPL, macular ganglion cell-inner plexiform layer; VF, visual field; MD, mean deviation; PSD, pattern standard deviation; dB, decibel; PERG, pattern electroretinogram. Data are mean ± standard deviation unless otherwise indicated. * Student’s *t*-test. ^†^ Chi-square test. Data are mean ± standard deviation unless otherwise indicated. Factors with statistical significance are shown in bold.

**Table 3:** Correlation coefficients for RNFL thickness and GCIPL thickness with pattern ERG and perimetry in total subjects.

	RNFL Thickness		GCIPL Thickness	
	r	*p* Value	r	*p* Value
P50 amplitude	0.093	0.445	0.236	0.052
N95 amplitude	0.041	0.738	0.093	0.448
SITA 24-2MD	0.356	0.002	0.240	0.049
SITA 24-2PSD	−0.269	0.024	−0.194	0.114
SITA 10-2MD	0.217	0.071	0.437	0.000
SITA 10-2PSD	−0.250	0.037	−0.459	0.000
Threshold sensitive points (center)	0.180	0.136	0.385	0.001
Threshold sensitive points (total)	0.191	0.113	0.381	0.001

Pearson’s product-moment correlation analysis was used.

**Table 4:** Correlation coefficients for RNFL thickness and GCIPL thickness with pattern ERG and perimetry after grouping by MD progression.

	RNFL Thickness				GCIPL Thickness			
	High MD		Low MD		High MD		Low MD	
	r	*p* Value	r	*p* Value	r	*p* Value	r	*p* Value
P50 amplitude	−0.073	0.678	0.202	0.245	0.107	0.540	0.347	0.048
N95 amplitude	−0.118	0.500	0.040	0.821	−0.038	0.830	0.141	0.432
SITA 24-2MD	−0.019	0.915	0.177	0.308	−0.013	0.942	0.181	0.313
SITA 24-2PSD	0.039	0.824	−0.152	0.382	−0.101	0.564	−0.108	0.551
SITA 10-2MD	−0.155	0.375	0.191	0.273	0.053	0.763	0.500	0.003
SITA 10-2PSD	−0.085	0.628	−0.216	0.212	−0.327	0.055	−0.466	0.006
Threshold sensitive points (center)	−0.039	0.826	0.131	0.455	0.062	0.724	0.441	0.010
Threshold sensitive points (total)	−0.051	0.773	0.110	0.531	0.072	0.683	0.430	0.013

Pearson’s product-moment correlation analysis was used.

**Table 5:** Correlation coefficients for SITA 10-2MD and 10-2PSD with functional examination results in total subjects.

	SITA 10-2MD		SITA 10-2PSD	
	r	*p* Value	r	*p* Value
SITA 24-2MD	0.390	0.001	−0.293	0.014
SITA 24-2PSD	−0.305	0.010	0.445	0.000
Threshold sensitive points (center)	0.921	0.000	−0.638	0.000
Threshold sensitive points (total)	0.942	0.000	−0.734	0.000
P50 amplitude	0.002	0.986	−0.062	0.611
N95 amplitude	0.125	0.301	−0.163	0.179

Pearson’s product-moment correlation analysis was used.

**Table 6:** Factors associated with PSD of VF 10-2.

Variables	Univariate		Multivariate	
	HR (95% CI)	*p* Value	HR (95% CI)	*p* Value
Age (y)	1.02 (0.97–1.08)	0.418		
Female, no. (%)	0.61 (0.09–4.02)	0.614	0.47 (0.21–1.04)	0.070
Axial length (mm)	0.92 (0.6–1.4)	0.700	0.63 (0.51–0.79)	0.0002
Central corneal thickness (μm)	0.99 (0.97–1.02)	0.610		
Average pRNFL thickness (μm)	0.93 (0.84–1.02)	0.131		
Average mGC/IPL thickness (μm)	0.81 (0.74–0.89)	<0.0001	0.90 (0.85–0.95)	0.001
MD of VF 24-2 (dB)	0.72 (0.49–1.05)	0.095	1.67 (1.32–2.13)	0.0001
PSD of VF 24-2 (dB)	1.82 (1.31–2.54)	0.001	1.43 (1.13–1.80)	0.009
MD of VF 10-2 (dB)	0.44 (0.37–0.51)	<0.0001	0.24 (0.17–0.33)	<0.0001
Threshold sensitive points (center)	0.55 (0.46–0.67)	<0.0001	1.96 (1.47–2.59)	<0.0001
Threshold sensitive points (total)	0.44 (0.36–0.54)	<0.0001		
P50 amplitude of PERG	0.85 (0.44–1.66)	0.640	0.80 (0.60–1.08)	0.149
N95 amplitude of PERG	0.70 (0.39–1.24)	0.225		

pRNFL, peripapillary retinal nerve fiber layer; mGC/IPL, macular ganglion cell-inner plexiform layer; VF, visual field; MD, mean deviation; PSD, pattern standard deviation; dB, decibel; PERG, pattern electroretinogram. Data are mean ± standard deviation unless otherwise indicated.

## Data Availability

The data that support the findings of this study are available on request from the corresponding author. The data are not publicly available due to privacy or ethical restrictions.
